# The DeltaN p63 Promotes EMT and Metastasis in Bladder Cancer by the PTEN/AKT Signalling Pathway

**DOI:** 10.1155/2022/9566055

**Published:** 2022-04-12

**Authors:** Yong-Hua Tian, Yun-Feng He, Jin-Si Tan, Yu Jiang, Qiao Xu, Hong-Lin Cheng

**Affiliations:** ^1^Department of Urology, The First Affiliated Hospital of Chongqing Medical University, Chongqing 400042, China; ^2^Department of Urology, Youyang Hospital, A Branch of The First Affiliated Hospital of Chongqing Medical University, Chongqing 409800, China

## Abstract

Bladder cancer is a common tumour of the urinary system, and more than 90% is urothelial carcinoma. Therefore, it is important for discovering the key target genes and molecules of bladder tumour cell metastasis and invasion. Our research initially explored the regulation of deltaN p63 on the progression and metastasis of bladder cancer and found that deltaN p63 can influence the occurrence of EMT through PTEN and ultimately regulate the growth and metastasis of bladder cancer. In summary, this study identified a new EMT regulator, deltaN p63, further revealed the mechanism of the invasion and metastasis of bladder cancer cells, and provided a theoretical basis for finding new target molecules and drugs to treat bladder cancer. In conclusion, this study will further reveal the mechanism of tumour cell invasion and metastasis and provide a theoretical basis for cancer treatment to find new target molecules and drugs.

## 1. Introduction

Bladder cancer is a common tumour of the urinary system, and more than 90% is urothelial carcinoma [1, 2]. The incidence of bladder cancer ranks fourth among male malignancies in the world and eighth among male malignancies in our country, and it has been gradually increasing in recent years [2]. Recurrence and metastasis after surgery are major difficulties that plague clinicians, and the tumour may be accompanied by an increase in the degree of malignancy, which increases the difficulty of treatment [3]. Therefore, discovering the key target genes and molecules of bladder tumour cell metastasis and invasion and further revealing the mechanism of tumour cell invasion and metastasis provides a theoretical basis and reference for the treatment of bladder tumours.

Current research on deltaN p63 and bladder tumours focuses on its role as an oncogene to promote tumorigenesis and its regulation [4]. There are currently few studies on deltaN p63 and bladder tumour invasion, metastasis, and EMT. A previous report by our research group on the preliminary research results of deltaN p63 and tumour invasion, metastasis, and cell adhesion function preliminarily confirmed that deltaN p63 can regulate the adhesion ability between bladder tumour cells and that downregulating the function of deltaN p63 can enhance the adhesion ability and related adhesion of bladder tumour cells [5]. Downregulating the function of deltaN p63 can enhance the adhesion ability of bladder tumour cells and the expression of the related adhesion protein F-actin, which may inhibit the invasion and metastasis of bladder tumours and the occurrence of EMT [6]. Many studies have shown that epithelial-mesenchymal transition (EMT) is closely related to the invasion and metastasis of tumour cells [7, 8]. Simultaneously, other studies have shown that epithelial-mesenchymal transition (EMT) is related to the refinement and refinement of tumour cells [7, 8]. An increasing number of studies have also demonstrated that EMT is the key initiation step of tumour metastasis and participates in a variety of the processes of tumour invasion and metastasis, including bladder cancer [9, 10]. EMT is regulated by a complex network of signalling pathways [11, 12]. An in-depth study of the occurrence of EMT in the malignant evolution of tumour cells will further explore the synergy of many influencing factors and may identify new regulatory factors, which will help to better understand the relationship between EMT and bladder cancer tumour occurrence and development.

Therefore, this study confirmed that deltaN p63 can promote the invasion and metastasis of bladder tumours and the occurrence of EMT by inhibiting the PTEN signalling pathway. This study will further reveal the mechanism of tumour cell invasion and metastasis and provide a theoretical basis for cancer treatment to find new target molecules and drugs.

## 2. Methods

### 2.1. Tissue Samples

A total of 42 paired bladder cancer and paired paracarcinoma normal tissues were collected from bladder cancer patients who had undergone resection between 2012 and 2018 at the Department of Urology, the First Affiliated Hospital of Chongqing Medical University. The specimens were snap-frozen after resection and stored in liquid nitrogen for further analysis. All involved patients provided written informed consent, and the study was approved by the Ethics Committee on Human Research of the hospital.

### 2.2. Cell Lines

A human bladder cancer cell line (5637) and the immortalized human foetal osteoblastic cell line hFOB1.19 were obtained from the Cell Bank of the Chinese Academy of Sciences (Shanghai, China). All cells were maintained in complete growth medium as recommended by the manufacturer.

### 2.3. RNA Sequencing Array

First, rRNAs in samples from the control and PM2.5 groups were removed. Then, the libraries for next-generation sequencing were prepared using the TruSeq RNA Sample Prep Kit (Illumina, USA). After enrichment and purification, the libraries were processed for sequencing by Wuhan Nuoruihua Biopharm Technology Co., Ltd. (Wuhan, China) according to an available protocol.

### 2.4. RNA Isolation and Quantitative Real-Time PCR

Total RNA was extracted from tissues and cultured cells using an RNAiso plus Kit (Takara, China) according to the manufacturer's instructions and reverse transcribed using Hifair III 1st Strand cDNA Synthesis SuperMix for qPCR (gDNA digester plus) Kit (Yeasen, China). cDNA was subjected to qRT-PCR using Hieff qPCR SYBR Green Master Mix (Yeasen, China) with a Roche Light Cycler 96 System (Roche Life Science, Switzerland) to detect deltaN p63 mRNA. GAPDH was selected as the endogenous control for deltaN p63. The results were analysed using the 2^−ΔΔCt^ methods. qRT-PCR was performed in triplicate. The primer sequences are given in Table 1.

### 2.5. Analysis of Cell Viability and Apoptosis

The Cell Counting Kit-8 (CCK-8, Dojindo, Japan) was used to evaluate cell viability. Briefly, cells were seeded into 96-well plates at 5000 cells/well and cultured. CCK-8 (1 : 10 dilution) was added to each well every 24 hours and incubated for 2 hours at 37°C, and then, the optical density at 450 nm was detected daily using a microplate reader (Bio-Rad, USA) [13].

For cell apoptosis analysis, 2 × 10^5^ cells were seeded in six-well plates and incubated with DDP (1 *μ*g/mL) for 48 hours, and the apoptosis level was analysed by flow cytometry. Briefly, cells were trypsinized, resuspended, and stained with a FITC Annexin V Apoptosis Detection Kit with PI (BD Biosciences, USA). Stained cells were analysed on a FACScan flow cytometer (BD Biosciences, USA). The results are presented as a percentage of apoptotic cells relative to total cells.

### 2.6. Transwell Assays

After 48 hours of transfection, 1 × 10^5^ cells in 200 *μ*L serum-free medium were plated into upper chambers coated with 50 *μ*L serum-free Matrigel (1 : 9, BD Biosciences, USA) in transwell inserts (8 *μ*m pore size, Corning, USA) in a 24-well plate. The lower chamber was filled with 500 *μ*L of complete medium. Cells were incubated for 48 hours. Then, the cells on the upper surface were wiped slightly using cotton swabs. Cells that invaded the lower surface were fixed with 4% paraformaldehyde, stained with 0.1% crystal violet, and counted under an inverted microscope (Nikon, Japan).

### 2.7. Immunohistochemistry (IHC)

IHC was performed as described previously [4]. Primary antibodies against deltaN p63, PTEN, and Ki67 were purchased from Abcam (USA).

### 2.8. Western Blot

A total of 30 *μ*g of protein was separated by 10% SDS-PAGE and transferred onto polyvinylidene fluoride membranes (Millipore, USA). The membranes were blocked with 5% skim milk in Tris-buffered saline-Tween (TBST) for 1 hour at room temperature and then incubated with primary antibodies against deltaN p63 (1 : 500), PTEN (1 : 800), E-cadherin (1 : 500), vimentin (1 : 500), Snail (1 : 1000) (Cell Signalling Technology, USA), and GAPDH (Proteintech, China) overnight at 4°C. Subsequently, the membranes were washed three times with TBST and probed with corresponding horseradish peroxidase-conjugated secondary antibodies (Proteintech, China) for 1 hour at room temperature. Proteins of interest were visualized using ECL Western blotting substrate (Pierce, USA) [8].

### 2.9. Statistical Analysis

Data were analysed from triplicate experiments and presented using GraphPad Prism 6.0 (GraphPad, USA) and SPSS 18.0 (IBM, USA). Statistical significance was considered when the *p* value was less than 0.05. All data are presented as the mean ± standard deviation (s.d.), and significant differences between each group were evaluated using Student's *t*-test or one-way ANOVA.

## 3. Results

### 3.1. Analysis of the Differential Expression of Genes in Metastatic and Nonmetastatic Bladder Cancer Tissues by Biosynthesis

**Figure 1:**
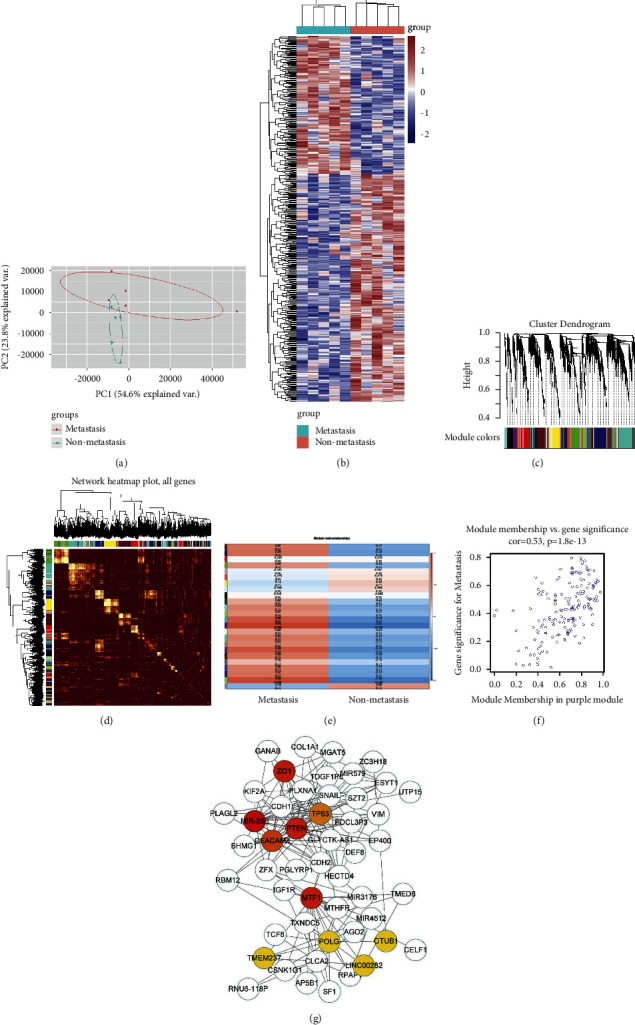
Bioinformatics analysis of the differential expression of genes in metastatic and nonmetastatic bladder cancer tissues. (a) The PCA dimensionality reduction analysis of the sequencing results. (b) The heat map display of the sequencing results. (c) The weights of these genes in the modules. (d) The correlation between these modules shown in WGCNA. (e)-(f) The correlation between different modules and groups. (g) PPI protein interaction network analysis.

The PCA dimensionality reduction analysis of the sequencing results showed significant differences between the two groups of tissues (Figure 1(a)).  Figure 1(b) shows a heatmap display of the sequencing results. The two sets of gene transcriptome expression differ greatly. According to the WGCNA algorithm, the sequencing results were analysed, the genes were divided into different modules, and then, the weights of these genes in the modules were displayed (Figure 1(c)). The correlation between these modules is shown in WGCNA (Figure 1(d)). The correlation between different modules and groups is shown in Figures 1(e) and 1(f). Finally, PPI protein interaction network analysis was used to calculate the hub genes in the purple module with Cytoscape. The expression of p63 is significantly different in the metastatic tissues of bladder cancer, and PTEN may be an important interacting gene of p63.

### 3.2. DeltaN p63 Is an Important Protein That Promotes the Progression of Bladder Cancer

**Figure 2:**
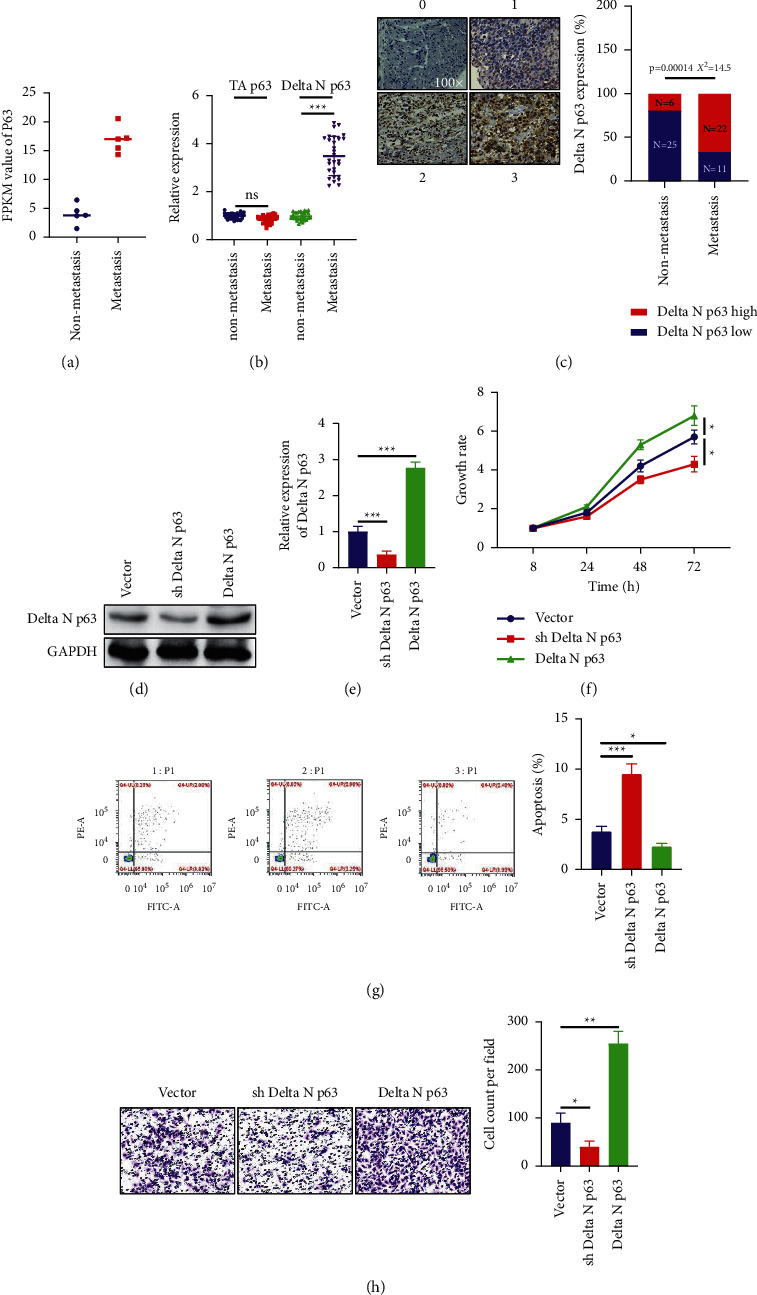
DeltaN p63 promotes the progression of bladder cancer. (a) RT-PCR detection of p63 expression in bladder cancer. (b) RT-PCR detection of two subtypes of p63 in bladder cancer tissues. (c) IHC detection of two subtypes of p63 in bladder cancer tissues. (d) WB detection of deltaN p63 protein expression after transfection. (e) RT-PCR detection of deltaN p63 protein expression after transfection. (f) CCK-8 analysis of cell growth after transfection. (g) Flow cytometric detection of apoptosis after transfection. (h) Transwell detection of metastasis after transfection.

To further verify the results of the bioinformatics analysis, the value of PCR showed that the transcription level of p63 was upregulated in patients with metastasis (Figure 2(a)). There are two subtypes of p63: TAp63 promotes Ras-induced senescence and inhibits the occurrence of cancer, while deltaN p63 can promote the growth of bladder cancer. Our further subtype analysis showed that deltaN p63 expression was elevated in metastatic bladder cancer tissues (Figures 2(b) and 2(c)), suggesting that deltaN p63 plays a major role. Then, deltaN p63 siRNA and plasmid were constructed, and after transfecting the cells, WB detection verified that the construction was successful (Figures 2(d) and 2(e)). CCK-8 analysis of cell growth showed that knocking down the expression of deltaN p63 can inhibit the growth and metastasis of bladder cancer cells and promote cell apoptosis, while overexpression of deltaN p63 can promote the growth and metastasis of bladder cancer cells but inhibit cell apoptosis (Figures 2(f)–2(h)).

### 3.3. EMT Is an Important Regulatory Pathway of DeltaN p63

**Figure 3:**
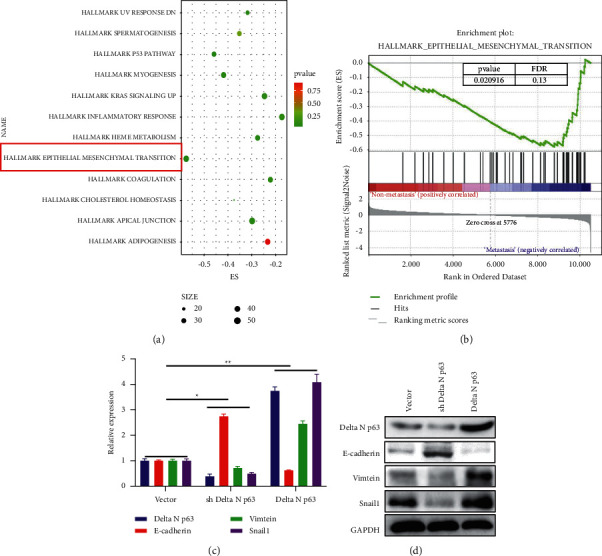
DeltaN p63 could regulate the EMT pathway. (a) KEGG analyzing the downstream regulatory pathways of deltaN p63. (b) GSEA analyzing the downstream regulatory pathways of deltaN p63. (c) RT-PCR detection of the EMT pathway mRNA level after knockdown or increased deltaN p63 expression. (d) WB detection of the EMT pathway mRNA level after knockdown or increased deltaN p63 expression.

We used sequencing results to analyse the downstream regulatory pathways of deltaN p63. Both KEGG and GSEA showed that EMT is an important downstream regulatory pathway of deltaN p63 (Figure 3(a) and 3(b)). Knockdown of deltaN p63 can prevent the activation of the EMT pathway by upregulating the expression of E-cadherin protein and mRNA and inhibiting the expression of vimentin and Snail. However, overexpression of deltaN p63 activated the EMT pathway by affecting the expression of E-cadherin, vimentin, and Snail (Figures 3(c) and 3(d)).

### 3.4. PTEN Is an Important Target Gene for DeltaN p63 to Regulate EMT

**Figure 4:**
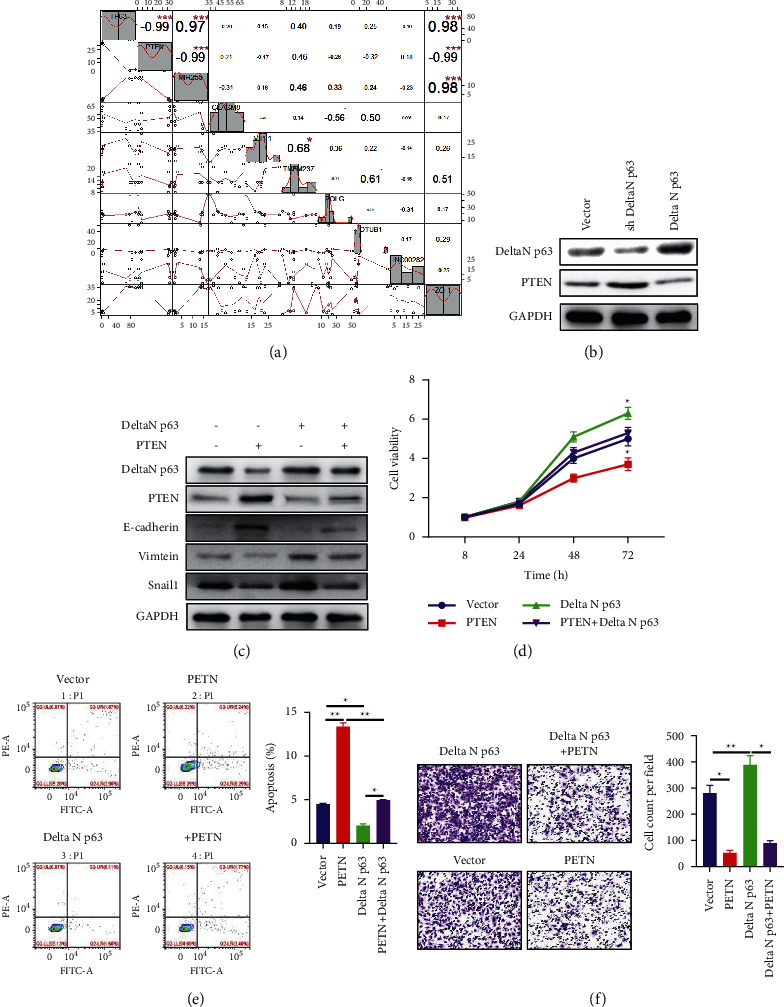
DeltaN p63 regulates EMT through PTEN. (a) R language toolkit to analyse the correlation between p63 and related gene expression. (b) WB detecting the expression of PTEN protein after knockdown or overexpress deltaN p63. (c) WB detecting the expression of EMT pathway protein after overexpression of deltaN p63 and/or PTEN. (d) CCK-8 analysis of cell growth after overexpression of deltaN p63 and/or PTEN. (e) Flow cytometric detection of apoptosis after overexpression of deltaN p63 and/or PTEN. (f) Transwell detection of metastasis after after overexpression of deltaN p63 and/or PTEN.

We used the R language toolkit to analyse the correlation between p63 and related gene expression and found that p63 was negatively correlated with PTEN expression (Figure 4(a)). We subsequently found through WB detection that knocking down deltaN p63 can upregulate the expression of PTEN protein and mRNA, and overexpression of deltaN p63 can inhibit PTEN expression (Figures 4(b) and 4(c)). Then, we used pathway blocking experiments. When deltaN p63 and PTEN were overexpressed simultaneously, PTEN blocked the activation of deltaN p63 in the related EMT pathway. The promotion effect of deltaN p63 on cell growth and metastasis was decreased (Figures 4(d)–4(f)), and the inhibitory effect on apoptosis was also decreased (Figure 4(e)).

### 3.5. DeltaN p63 Regulates the Growth of Bladder Cancer through Targeting PTEN In Vivo

**Figure 5:**
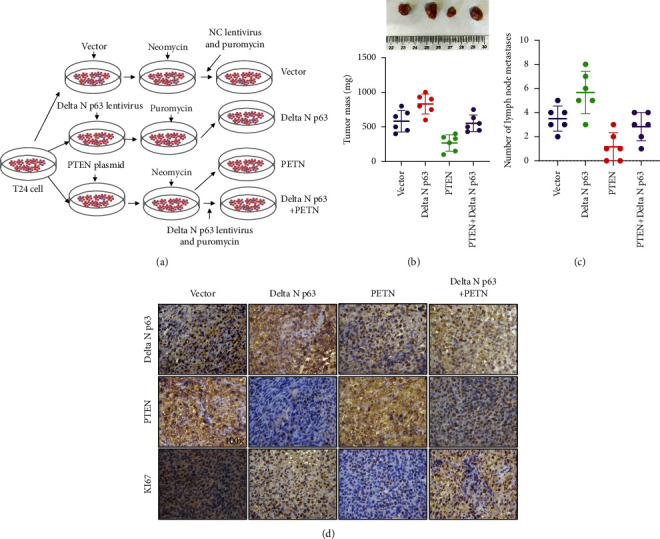
DeltaN p63 promote the growth of bladder cancer by target PTEN in vivo. (a) Animal experiment flowchart. (b) Tumour weight. (c) Number of lymph node metastasis. (d) IHC detecting the expression of deltaN p63, PTEN, and Ki67.

To understand the effect of deltaN p63 on the metastasis of bladder cancer in vivo, we first constructed a cell line stably expressing deltaN p63 and PTEN (Figure 4(a)). The cells were injected into the mouse bladder to form tumours in situ. DeltaN p63 can promote the growth of bladder cancer (Figure 5(b)) and lymph node metastasis (Figure 5(c)), but PTEN can inhibit the above changes caused by deltaN p63. Immunohistochemical detection revealed that PTEN can inhibit the expression of Ki67, a proliferation protein induced by deltaN p63 (Figure 5(d)).

## 4. Discussion

Recent studies have found that deltaN p63 and miR-205 are related in prostate tissue. When deltaN p63 expression is silenced, the expression of miR-205 also decreases, and when deltaN p63 is overexpressed, miR-205 expression is enhanced [7]. Current research on deltaN p63 and bladder tumours focuses on its role as an oncogene to promote tumorigenesis and its regulation. Our study found that EMT is an important regulatory pathway for deltaN p63 through bioinformatics analysis and a series of in vivo and in vitro experiments. Downregulation of deltaN p63 can inhibit the invasion and metastasis of bladder tumours and the occurrence of EMT, consistent with the research results of Zhao in cervical squamous cell carcinoma.

EMT is regulated by a complex network of signalling pathways. EMT leads to nonmigratory cells gaining the ability to infiltrate and eventually metastasize to other tissues and organs, thereby causing the tumour to form distant metastases. Tumour cells undergo EMT to transition from a benign to a malignant phenotype [14].

We further found that PTEN is the key gene by which deltaN p63 regulates EMT and metastasis through expression correlation analysis and in vivo and in vitro experiments. PTEN is a tumour suppressor gene with bispecific phosphatase activity, and it has a broad relationship with the p53 gene. PTEN plays important roles in the process of shedding particles, circulation, and formation of tumour metastases [15, 16]. However, there are still many important questions about PTEN and its signal transduction pathways that are still unclear. This study found that deltaN p63 regulates EMT through PTEN and affects the invasion and metastasis of bladder cancer through in vivo and in vitro experiments, which explored a new signal transduction pathway for PTEN.

In summary, our research initially explored the regulation of deltaN p63 on the progression and metastasis of bladder cancer and found that deltaN p63 can influence the occurrence of EMT through PTEN and ultimately regulate the growth and metastasis of bladder cancer. This study identified a new EMT regulator, deltaN p63, further revealed the mechanism of the invasion and metastasis of bladder cancer cells, and provided a theoretical basis for finding new target molecules and drugs to treat bladder cancer.

## Figures and Tables

**Table 1:** The primer sequences.

	Sequence (5′-3′)
DeltaN p63	
Forward	5′-TGC CCA GAC TCA ATT TAG TGA G-3′
Reverse	5′-TCT GGA TGG GGC ATG TCT TTG C-3′
Snail	
Forward	5′-TTACCTTCCAGCAGCCCTAC-3′
Reverse	5′-GCTTCGGATGTGCATCTTG-3′
E-cadherin	
Forward	5′-ACCCCCACTGAAAAAGATGA-3′
Reverse	5′-GCATCTTCAAACCTCCATGAT-3′
Vimentin	
Forward	5′-AATGGCTCGTCACCTTCG-3′
Reverse	5′-CTAGTTTCAACCGTCTTAATCAG-3′
GAPDH	
Forward	5′-AGAAGGCTGGGGCTCATTTG-3′
Reverse	5′-AGGGGCCATCCACAGTCTTC-3′

## Data Availability

The datasets used and/or analysed during the present study are available from the corresponding author upon request.
